# ΔRR vaccination protects from KA-induced seizures and neuronal loss through ICP10PK-mediated modulation of the neuronal-microglial axis

**DOI:** 10.1186/1479-0556-6-1

**Published:** 2008-01-07

**Authors:** Jennifer M Laing, Laure Aurelian

**Affiliations:** 1 Department of Pharmacology and Experimental Therapeutics, University of Maryland, School of Medicine, Baltimore, MD 21201, USA

## Abstract

Ischemic brain injury and epilepsy are common neurodegenerative diseases caused by excitotoxicity. Their pathogenesis includes microglial production of inflammatory cytokines. Our studies were designed to examine whether a growth compromised HSV-2 mutant (ΔRR) prevents excitotoxic injury through modulation of microglial responses by the anti-apoptotic HSV-2 protein ICP10PK. EOC2 and EOC20 microglial cells, which are differentially activated, were infected with ΔRR or the ICP10PK deleted virus (ΔPK) and examined for virus-induced neuroprotective activity. Both cell lines were non-permissive for virus growth, but expressed ICP10PK (ΔRR) or the PK deleted ICP10 protein p95 (ΔPK). Conditioned medium (CM) from ΔRR-, but not ΔPK-infected cells prevented N-methyl-D-aspartate (NMDA)-induced apoptosis of primary hippocampal cultures, as determined by TUNEL and caspase-3 activation (76.9 ± 5.3% neuroprotection). Neuroprotection was associated with inhibition of TNF-α and RANTES and production of IL-10. The CM from ΔPK-infected EOC2 and EOC20 cells did not contain IL-10, but it contained TNF-α and RANTES. IL-10 neutralization significantly (p < 0.01) decreased, but did not abrogate, the neuroprotective activity of the CM from ΔRR-infected microglial cultures indicating that ICP10PK modulates the neuronal-microglial axis, also through induction of various microglial neuroprotective factors. Rats given ΔRR (but not ΔPK) by intranasal inoculation were protected from kainic acid (KA)-induced seizures and neuronal loss in the CA1 hippocampal fields. Protection was associated with a significant (p < 0.001) increase in the numbers of IL-10+ microglia (CD11b+) as compared to ΔPK-treated animals. ΔRR is a promising vaccination/therapy platform for neurodegeneration through its pro-survival functions in neurons as well as microglia modulation.

## Introduction

Ischemic brain injury, or stroke, and epilepsy are two of the most common neurodegenerative disease in Americans, the symptoms of which are caused by excitotoxicity [1,2]. Excitotoxicity is a mechanism of neuronal cell injury that is caused by the excessive activation of glutamate receptors and is accompanied by the induction of neuronal cell apoptosis, a tightly regulated, energy dependent, irreversible process mediated by cysteine proteases (caspases) [3]. Microglia activation and the production of inflammatory cytokines, namely TNF-α, were associated with neurodegeneration, including excitotoxic injury [4-6]. Several strategies were proposed to interrupt the apoptotic cascade in neurons, including gene therapy with growth factors or anti-apoptotic proteins delivered by the neurotropic herpes simplex virus type 1 (HSV-1) [7,8]. However, these genes had relatively narrow neuroprotective profiles, neuronal survival was often limited and did not correlate with retention of functional integrity, and some strategies were associated with detrimental outcomes [8,9] potentially related to their effect on glial cells. Indeed, microglia are considered the CNS resident professional macrophages. They function as the principal immune effector cells of the CNS, responding to any pathological event. Activated microglia accumulate at sites of injury or plaques in neurodegenerative CNS, and their activation was implicated in the pathogenesis of a variety of neurodegenerative diseases, including Alzheimer disease, Parkinson's disease, HIV-associated dementia and stroke. Excessive microglial activation and the dysregulated overproduction of inflammatory cytokines are the hallmark of many neurodegenerative diseases and ischemic brain injury [4-6,10,11]. Given their importance in modulating neuronal cell life/death decisions, microglia are increasingly recognized as a potential target for neuroprotective vaccination. However, identification of the correct gene for vaccine development is a major clinical challenge. We have recently described the construction of a growth compromised HSV-2 based vector (ΔRR) for the viral protein ICP10PK, which has anti-apoptotic activity in primary and organotypic hippocampal and striatal cultures through activation of survival pathways [12-18]. The studies described in this report were designed to examine whether ΔRR can function as a vaccine to prevent neurodegenerative injury through ICP10PK-mediated modulation of the microglial cell responses in favor of neuroprotection.

## Materials and methods

### Cell culture

Vero (African green monkey kidney), SK-NSH (human neuroblastoma) and LADMAC (mouse bone marrow) cells were grown in minimal essential medium (MEM), supplemented with 1 mM sodium pyruvate, 2 mM L-glutamine, 100 μM non-essential amino acids and 10% fetal bovine serum (FBS) (Gibco-BRL, Gaithersburg, MD). EOC20 and EOC2 microglia cultures were obtained from ATCC (Manassas, VA) and grown in Dulbecco's minimal essential medium (DMEM, Gibco-BRL) with 20% 7 day-conditioned LADMAC medium which provides CSF-1 for microglial cell growth. EOC20, but not EOC2, cells constitutively express high levels of MHCII antigens [19]. Rat embryonic day 18 hippocampi were purchased from Neuromics (Edina, MN) and dissociated and plated at a density of 5 × 10^5 ^cells/dish on glass coverslips precoated with poly-L-Lysine (Sigma, St. Louis, MO) according to manufacturer's instruction. Over 99% of the cells stained with β_III_Tubulin antibody, indicating that they are neurons. The cultures were maintained in Neurobasal medium (Gibco-BRL) supplemented with B27 (Gibco-BRL).

### Viruses

**Figure 1:**
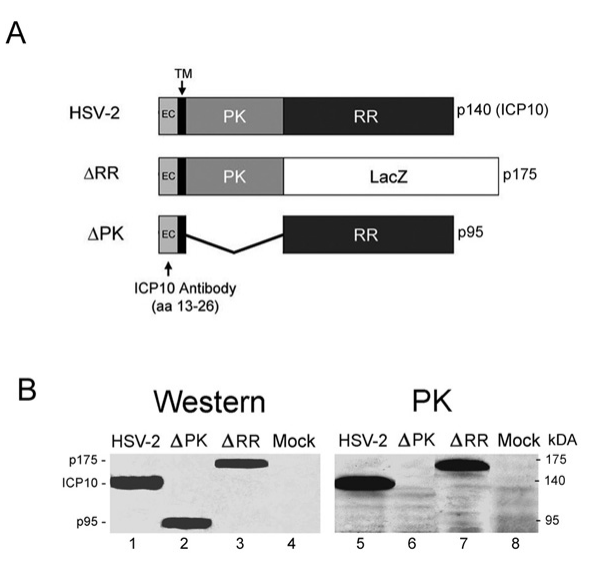
**Expression and kinase activity of mutant ICP10 proteins**. (A). Schematic representation of the ICP10 and mutant proteins. The wild type ICP10 protein expressed by HSV-2 is a 140 kDa chimera that contains an amino-terminal PK domain and a carboxy-terminal RR domain. In ΔRR, the RR domain was replaced with the β-galactosidase gene (LacZ) which was fused in frame to the PK domain, giving rise to a 175 kDa protein (p175). In ΔPK, the PK domain of ICP10 was deleted giving rise to a 95 kDa protein (p95). All three proteins (ICP10, p175 and p95) retain the transmembrane (TM) and extracellular (EC) domains and amino acids 13–26, which are recognized by the ICP10 antibody. (B). SK-NSH cells infected with HSV-2, ΔRR, ΔPK or PBS (mock-infected) were collected at 18 hrs after infection and cell extracts were assayed for protein expression (western) and ICP10 kinase activity (PK) using immunoblotting and immunocomplex kinase assays with ICP10 antibody.

HSV-2 (strain G) and the mutants ΔPK and ΔRR constructed from HSV-2(G) were previously described [12-14,16-18,20,21]. Briefly, to construct ΔRR, we took advantage of previous findings that the large subunit of the HSV-2 ribonucleotide reductase (R1, also known as ICP10), which is encoded by the viral gene UL39, has independently functioning protein kinase (ICP10PK) and ribonucleotide reductase (RR) domains, both of which are required for virus growth in non-replicating cells, including neurons [17,18,20]. To generate ΔRR, the 3'-end R1-encoding sequences of UL39 were deleted and replaced with LacZ fused in frame with ICP10PK, giving rise to a 175 kDa mutant protein (p175). ΔPK was generated from ΔRR by deletion of the UL39 5'-end sequences that encode ICP10PK giving rise to a 95 kDa protein (p95) (Fig. 1A). Expression of the p175 and p95 proteins is driven by the authentic ICP10 promoter, which is regulated with immediate early (IE) kinetics (independent of virus replication) and responds to AP-1 transcription factors upregulated/activated by neurotoxic stress stimuli [22-24]. ΔRR and ΔPK are grown in Vero cells and titrated by plaque assay in medium containing 10% serum [20].

### Antibodies and reagents

The generation and specificity of the rabbit ICP10 antibody was described. It recognizes an epitope located within amino acid residues 13–26 that are retained by both p175 and p95 [13,14,17,18,20,21]. The following antibodies were purchased and used according to the manufacturer's instructions: CD11b (Mac-1α_m _chain-biotin conjugated; Leinco, St. Louis, MO), HSV major capsid protein VP5 (Virusys Corporation, Sykesville, MD), TNF-α and neutralizing IL-10 (R&D Systems, Minneapolis, MN), IL-10 (Santa Cruz Biotechnology, Santa Cruz, CA), p20 fragment of activated caspase-3 (caspase-3p20) (Cell Signaling Technologies, Beverly, MA) and β_III_Tubulin (Promega, Madison, WI). Texas Red conjugated streptavidin, FITC conjugate streptavidin, Texas Red conjugated horse anti mouse IgG and FITC conjugated goat anti rabbit was purchased from Vector (Burlingame, CA), FITC conjugated goat anti mouse IgG from Jackson ImmunoResearch (West Grove, PA), AlexaFluor 546 was purchased from Molecular Probes (Eugene, OR), *N*-methyl-D-aspartic acid (NMDA) from Sigma-Aldrich, and Kainic Acid (KA) from A.G. Scientific (San Diego, CA).

### Immunoblotting and immunocomplex PK assay

Immunoblotting was performed as described [19,23]. Briefly, cells were lysed with radioimmunoprecipitation buffer [RIPA; 20 mM Tris-HCl (pH 7.4), 0.15 mM NaCl, 1% Nonidet P-40, 0.1% sodium dodecyl sulfate (SDS), 0.5% sodium deoxycholate] supplemented with protease and phosphatase inhibitor cocktails (Sigma) and sonicated twice for 30 seconds at 25% output power with a Sonicator ultrasonic processor (Misonix, Inc., Farmingdale, NY). Protein concentrations were determined by the bicinchoninic assay (Pierce, Rockford, IL), and 100 μg protein samples were resolved by SDS-polyacrylamide gel electrophoresis (SDS-PAGE) and transferred to nitrocellulose membranes. The blots were incubated (1 hr, RT) in TNT buffer (0.01 M Tris-HCl [pH 7.4], 0.15 M NaCl, 0.05% Tween 20) containing either 5% nonfat dried milk or 1% bovine serum albumin (BSA) to block nonspecific binding. Blots were exposed overnight at 4°C to appropriate antibodies diluted in TNT buffer with either milk or BSA, washed in TNT buffer, and incubated (1 hr; RT) with anti-rabbit IgG conjugated to horseradish peroxidase (HRP; Cell Signaling). After extensive washing, bands were detected using enhanced chemiluminescence reagents (ECL, Amersham Pharmacia, Piscataway, NJ) and exposure to high-performance film (Hyperfilm ECL, Amersham). Quantitation was by densitometric scanning with the Bio-Rad GS-700 imaging densitometer (Bio-Rad, Hercules, CA) and results are expressed as densitometric units × 100. For immunocomplex PK assays cell extracts in lysis buffer (20 mM Tris, pH 7.5, 150 mM NaCl, 1% NP-40 and protease and phosphatase inhibitor cocktails) were standardized for protein concentration and incubated with 10 μl of ICP10 antibody (1 h, 4°C) and 100 μl of protein A-sepharose CL4B beads (50% v/v) (30 min, 4°C). The beads were washed (3×) with RIPA buffer followed by TS buffer [20 mM Tris-HCl (pH 7.4), 0.15 M NaCl], resuspended in 50 μl kinase reaction buffer consisting of 10 μCi [32P]-ATP (0.1 μM, 3000 Ci/mmol, NEN), 5 mM MgCl_2_, 2 mM MnCl_2_, 20 mM Tris-HCl (pH 7.4), and incubated at 30°C for 30 min. Samples were washed in 20 mM Tris-HCl (pH 7.4) with 0.15 M NaCl and boiled for 5 min after addition of 100 μl denaturing solution. Proteins were resolved by SDS-PAGE.

### Single step growth curves and infectious centers assay

Single step growth curves were done as described [13,18,20]. Infection was with 5 plaque forming units (pfu)/cell and adsorption was for 2 hrs at 37°C (0 hrs in growth curve). Three cultures/time point were harvested and virus titers, determined by plaque assay. For infectious center assays, microglia (200 or 500 cells) were plated on Vero cells and plaques were counted 48 hrs later. Results are expressed as % infectious centers = (mean No. plaques/No. plated cells) × 100.

### TUNEL, immunofluorescence and LacZ expression

The *In situ *Cell Death Detection kit (Roche) was used for TUNEL assays, according to the manufacturers' instructions. Briefly, cells grown on glass slides were fixed in 4% paraformaldehyde in PBS, pH 7.4 [1 hr, room temperature (RT)] followed by permeabilization in 0.1% Triton-X (in 0.1% sodium citrate) for 2 minutes on ice. DNA breaks were labeled by incubation (60 min; 37°C) with terminal deoxynucleotidyl transferase and nucleotide mixture containing flourescein isothiocyanate (FITC)-conjugated dUTP (TUNEL reagent). Cells were then washed with PBS and mounted in Vectashield with DAPI (Vector, Burlingame, CA) and visualized. %. For immunofluorescent staining, cells were permeabilized with 0.1% Triton X-100 [in 0.1% sodium citrate buffer (2 min; RT)] and blocked with 5% normal goat serum and 5% BSA (30 min; RT). They were incubated with primary antibody (18 hrs; 4°C), washed in PBS with 0.1% Tween 20 and exposed to fluorochrome labeled secondary antibodies (1 hr; 37°C). Slides were mounted in Vectashield with DAPI (Vector) and visualized as before. To determine expression of ICP10PK (p175), EOC2, EOC20, and Vero cells were infected with 5 pfu/cell of ΔRR and the infection was synchronized by adsorption (1 hr) at 4°C followed by culture shift to 37°C (0 hrs p.i). Live cells that express the p175 protein were identified by staining with the green fluorescent β-galactosidase substrate, C_12_-fluorescein di-β-D-galactopyranoside (C_12_FDG; Molecular Probes) according to the manufacturer's instructions. Because ICP10PK is fused in frame with LacZ, C_12_FDG staining reflects ICP10PK expression [13]. Visualization was done with a Nikon E4100 fluorescent microscope utilizing FITC (330–380 nM), UV (for DAPI) (465–495 nM) and Texas Red (540–580 nM) cubes. Each experiment was done in triplicate and the % staining cells was determined by counting 5 randomly selected fields, (at least 250 cells each, in a 3 mm^2 ^area) and results are expressed as % positive cells/total number of cells determined by DAPI staining [13,14,17,21].

### Collection of microglia culture supernatants (CM) and ELISA

Culture supernatants (conditioned media, CM) were obtained from infected or mock infected EOC2 and EOC20 cultures (moi = 5; 48 hrs) and cleared of cell debris by centrifugation at 14,000 × g for 30 min. Although they were virus-free by plaque assay, the CM were exposed to ultraviolet light using a Sylvania G15 T8 bulb at a distance of 17 cm (30 min; room temperature) in order to insure virus inactivation, as previously described [18]. They were assayed by ELISA for TNF-α, RANTES (R&D Systems, Minneapolis, MN) and IL-10 (eBioscience, San Diego, CA), according to manufacturer's instructions.

### CM-mediated neuroprotection in culture

Hippocampal cultures were treated (or not) with NMDA (50 μM; 3 hrs), extensively washed and grown in a 1:1 mixture of Neurobasal medium supplemented with B27 and CM. CM in which IL-10 was neutralized by incubation (1 hr; 37°C) with 20 μg/ml of IL-10 antibody (R&D Systems) were studied in parallel. Neuroprotection was calculated according to the formula: % neuroprotection = [NMDA-(CM-B)/NMDA] × 100, where NMDA is the % caspase3-p20+ cells in cultures given NMDA alone, CM is the % caspase3-p20+cells in cultures incubated with CM, and B is the %caspase3-p20+ cells in untreated cultures (background).

### ΔRR vaccination and neuroprotection

Sprague Dawley male rats (8–10 weeks old) were obtained from Charles River Laboratories (Wilmington, MA, USA). Animals were housed on a 12 h light/dark cycle with water and food supplied ad libitum. All procedures were performed in accordance with the University of Maryland, Baltimore Institutional Animal Care and Use Committee. They were vaccinated with ΔRR [50 μl (2.5 × 10 ^6 ^pfu)] by intranasal instillation, using ΔPK or PBS as controls. Delivery was over 15 minutes with 1 min breaks between instillation into each naris. Three inoculations were given at 24 hour intervals, with the last instillation considered day 0 p.i. KA (A.G. Scientific, San Diego, CA) was administered 24 hrs later (day 1) by i.p. injection. The route and dose (15 mg/kg) of KA administration were previously shown to elicit a well-characterized seizure activity followed by cell loss in the hippocampus [25,26]. Clinical response was scored as an average behavioral score for each animal every hour using the previously defined scale: 0, normal; 1, catatonic staring and immobilization; 2, 'wet-dog shakes', abnormal ambulation, stretching of limbs; 3, rearing and falling behavior; 4, tonic-clonic seizure activity; 5, death [27]. Results are expressed as the mean behavioral score/hour for each treatment group ± SEM. In addition, the % animals in each treatment group that experienced tonic-clonic seizure activity (score = 4) was recorded for each hour. To asses neuronal cell loss in the hippocampus, brain sections were fixed with 4% PAF in PBS (30 min; RT) and stained with thionin (J.T. Baker, Phillipsburg, NJ, USA) for 30 min. Sections were dehydrated and mounted in Permount (Fisher Scientific, Fair Lawn, NJ, USA). The numbers of neurons were counted in 3 randomly selected CA1 fields of 29 μm^2 ^(at least 250 cells) from 5 serial sections for all animals and the data are expressed as % neuronal loss ± SEM relative to untreated brains.

### Statistical analyses

Analysis of variance (ANOVA) was performed with Sigma Stat version 3.1 for Windows (Systat Software, Point Richmond, CA)

## Results

### ΔRR and ΔPK express the mutant ICP10 proteins p175 and p95 but only p175 has kinase activity

ICP10 is a 140 kDa protein that consists of an amino-terminal domain, which has protein kinase (PK) activity and a carboxy-terminal domain, which has RR activity. The PK domain is preceded by a transmembrane (TM) domain and a short extracellular (EC) domain that retains amino acids 13–26, which are recognized by the ICP10 antibody [20]. In ΔRR, the RR domain of ICP10 was replaced with LacZ, which was fused in frame with ICP10PK, giving rise to a 175 kDa protein (p175). p175 retains the TM and EC domains of the wild type ICP10 protein and it is under the direction of the authentic ICP10 promoter. In ΔPK, the PK domain of ICP10 was deleted, giving rise to a 95 kDa protein (p95), which also retains the authentic EC and TM domains and is driven by the same wild type ICP10 promoter [20] (Fig. 1A).

SK-NSH cells (derived from neuroblastoma) were infected with ΔRR, ΔPK or HSV-2 and cell extracts obtained at 18 hrs post infection (p.i) were immunoblotted with ICP10 antibody. A 140-kDa protein, consistent with the wild type ICP10 [20], was seen in HSV-2 infected cells (Fig. 1B, lane 1). In cells infected with ΔPK, the antibody recognized a 95-kDa protein (p95) (Fig. 1B, lane 2) and in cells infected with ΔRR, it recognized a 175-kDa protein (p175) (Fig. 1A, lane 3). Mock-infected cells were negative (Fig. 1B, lane 4). Immunocomplex PK assays with ICP10 antibody identified a 140-kDa phosphorylated protein consistent with the autophosphorylated ICP10 in HSV-2 infected cells (Fig. 1B, lane 5). Kinase activity was retained by p175, which was also autophosphorylated (Fig. 1B, lane 7). p95 was kinase negative, as evidenced by the absence of phosphorylated proteins in the ΔPK-infected cells (Fig. 1B, lane 6). Phosphorylated proteins were not seen in immunocomplex PK assays of extracts from mock-infected cells (Fig. 1B, lane 8). The data support previous conclusions that the PK and RR domains of ICP10 function independently of each other [20], and confirm that the p175 protein expressed by ΔRR retains the ICP10 kinase activity.

### Microglia are non-permissive for virus growth

**Figure 2:**
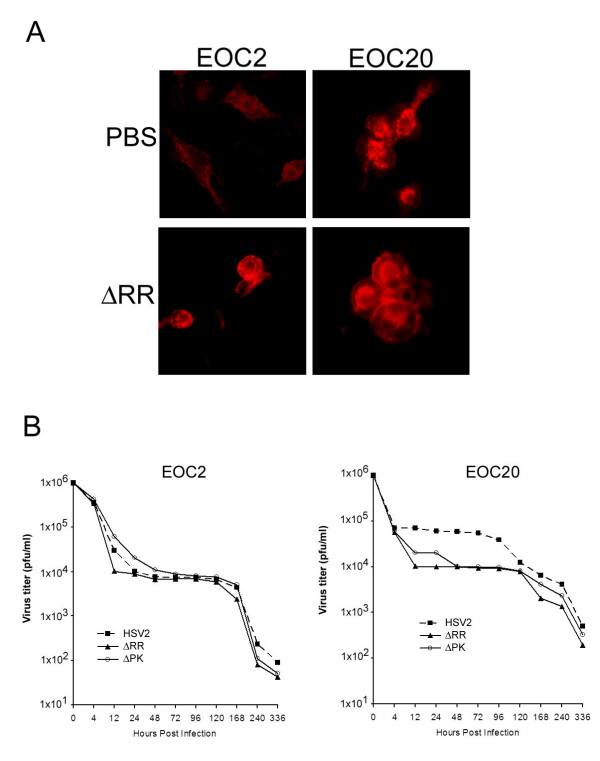
**EOC2 and EOC20 Microglia cultures are non-permissive for HSV replication**. (A). EOC2 and EOC20 cells differ in morphology and the intensity of staining with CD11b antibody before, but not after virus infection. (B). EOC2 and EOC20 cells were infected with ΔRR or ΔPK or HSV-2 (1 × 10^6 ^pfu) and examined for virus growth by plaque assay as described in Materials and Methods.

In a first series of experiments to examine the effect of ΔRR on microglia, we asked whether: (i) microglial cells are permissive for virus growth, and (ii) permissiveness is affected by prior cell activation. We used EOC2 and EOC20 cells that differ in the levels of MHCII expression, with high levels constitutively expressed by EOC20, but not EOC2 cells [19]. Excessive activation was confirmed for EOC20 cells by their rounded morphology and high intensity staining with CD11b antibody (Fig. 2A). EOC2 cells had lower CD11b staining intensity and retained some morphologic ramification. However, high intensity staining and rounded morphology were seen after virus infection (Fig. 2A), indicative of virus-induced activation [10,28]. EOC2 and EOC20 cells were non-permissive for growth of ΔRR, ΔPK or HSV-2, as determined by plaque assay. Virus titers decreased at similar rates during the first 4 hrs p.i.. For HSV-2, the titers remained at this reduced level until 96 hrs p.i. For ΔRR and ΔPK the titers continued to decrease until 12 hrs p.i. and remained stable at this reduced level until 120 hrs p.i. During 4 – 96 hrs p.i., the titers of ΔRR and ΔPK were approximately 10-fold lower than those of HSV-2, but virus clearance after 120 hrs was similar for all viruses, with lowest titers (almost complete clearance) seen at 14 days p.i. (Fig. 2B). Infectious center assays done up to 96 hrs p.i., indicated that approximately 90% of the cells formed plaques on Vero cells. Collectively, the data indicate that: (i) microglia are non-permissive for virus growth unrelated to their activation status prior to infection, and (ii) the clearance of ΔRR and ΔPK is somewhat more efficient than that of wild type virus.

### ICP10PK is expressed in ΔRR-infected EOC2 and EOC20 cells

**Figure 3:**
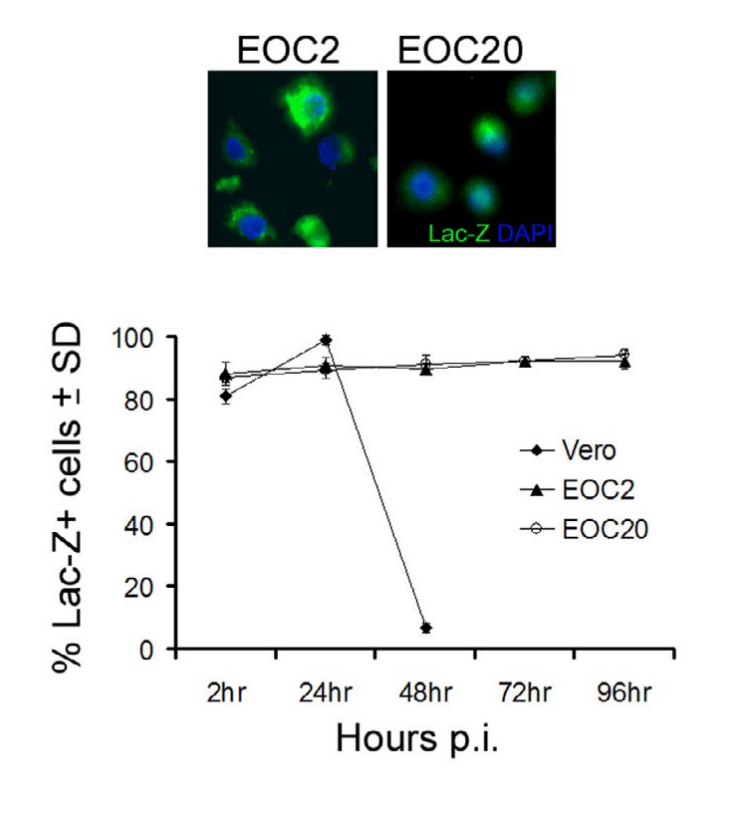
**ΔRR infected cells express ICP10PK**. (A) EOC2 and EOC20 cells infected with ΔRR (moi = 5) were stained with the LacZ substrate C_12_FDG at 24 hrs p.i. to visualize ICP10PK expression (Lac-Z). (B) EOC2, EOC20 and Vero cells were stained with C_12_FDG and the % staining cells at 4–96 hrs p.i. was determined by counting 5 randomly selected fields, (at least 250 cells each, in a 3 mm^2 ^area). Results are expressed as % positive cells/total number of cells determined by DAPI staining. The mean % ICP10PK (Lac-Z) ± SD are shown.

ICP10PK expression is regulated with IE kinetics and is independent of other viral proteins [22-24]. To verify that it is expressed in ΔRR-infected microglia. EOC2 and EOC20 cells were infected with 5 pfu/cell of ΔRR and the infection was synchronized as described in Materials and Methods. Vero cells, which are routinely used for virus growth, were studied in parallel as control for the effect of virus replication on ICP10PK expression. ICP10PK expression was determined by staining with the Lac-Z substrate C_12_FDG, as described [13]. In both EOC2 and EOC20 cultures, C_12_FDG staining was seen in most (90–95%) cells at 2–96 hrs pi. In Vero cells, C_12_FDG staining was seen in 80–97% of the infected cells at 2–24 hrs p.i., but expression was lost by the end of the replicative cycle (Fig. 3). The data indicate that ICP10PK expression is sustained in ΔRR-infected microglial cells for a relatively long time, and it is independent of the cell activation state.

### ΔRR does not trigger apoptosis in EOC2 and EOC20 cells

**Figure 4:**
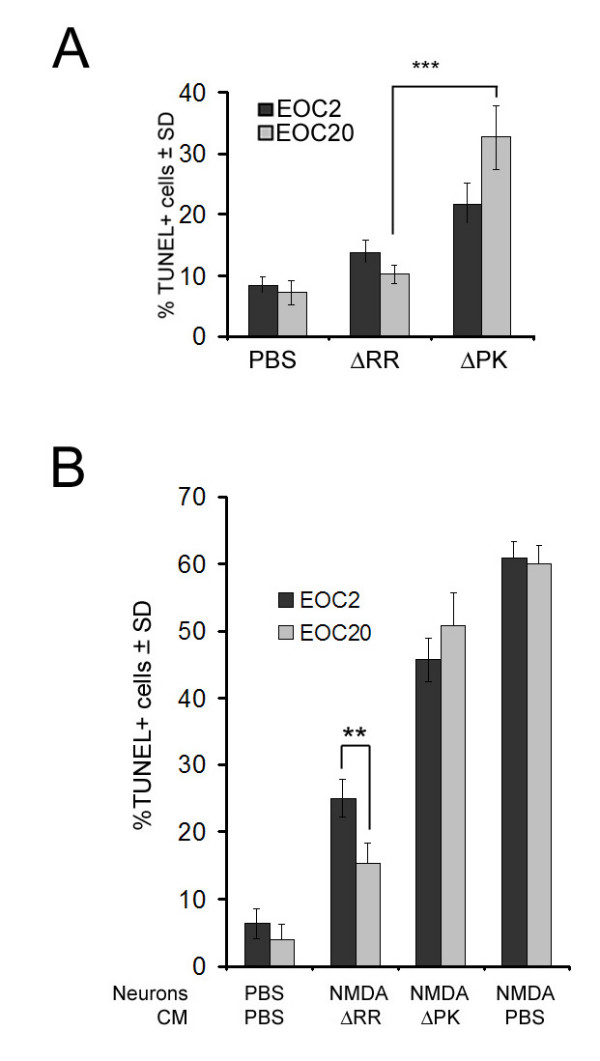
**ICP10PK inhibits apoptosis in ΔRR-infected microglia and CM from the infected microglia have neuroprotective activity**. (A). EOC2 and EOC20 cells were infected with ΔRR or ΔPK (moi = 5) or mock infected with PBS, and assayed for apoptosis by TUNEL at 24 hrs p.i. Each experiment was done in triplicate and the % staining cells was determined by counting 5 randomly selected fields, (at least 250 cells each, in a 3 mm^2 ^area). Results are expressed as % TUNEL+ cells/total number of cells determined by DAPI staining. The mean TUNEL+ cells ± SD are shown (***p < 0.001 relative to mock). (B). EOC2 and EOC20 cells were mock infected with PBS or infected with ΔRR or ΔPK (moi = 5) and culture supernatants (CM) were collected at 48 hrs p.i. and UV-treated as described in Materials and Methods. Primary hippocampal neurons treated (3 hrs) with NMDA (50 μM) or PBS, were extensively washed with MEM and re-incubated with a mixture (1:1) of Neurobasal medium containing B27 and CM from the infected microglia. They were fixed 24 h later and assayed for cell death by TUNEL. Each experiment was done in triplicate and the % staining cells was determined by counting 5 randomly selected fields, (at least 250 cells each, in a 3 mm^2 ^area). Results are expressed as % TUNEL+ cells/total number of cells determined by DAPI staining. The mean TUNEL+ cells ± SD are shown (**p < 0.01).

Having seen that ICP10PK is expressed in microglia, we wanted to know whether it inhibits virus-induced apoptosis. EOC2 and EOC20 cells were infected with ΔRR or ΔPK (moi = 5) or mock-infected with PBS and examined for apoptosis by TUNEL at 24 hrs p.i. The % TUNEL+ (apoptotic) cells were minimal in mock-infected EOC2 and EOC20 cells (9.3 ± 1.5 and 8.7 ± 1.9%, respectively). Infection with ΔPK caused a significant (p < 0.001) increase in the % TUNEL+ cells (32.6 ± 5.2 and 21.8 ± 3.3% for EOC20 and EOC2, respectively), but this increase was not seen in ΔRR-infected cells (13.8 ± 1.9 and 10.2 ± 1.4% for EOC2 or EOC20, respectively) (Fig 4A). The data indicate that ICP10PK overrides virus-induced microglial cell apoptosis independent of the state of cell activation prior to infection.

### CM from ΔRR infected EOC2/EOC20 cells protect hippocampal neurons from excitotoxin-induced apoptosis

In response to injury and neuronal stress/apoptosis, microglia in the surrounding area are activated and release inflammatory cytokines, which perpetuate cell death [29]. However, signals released by apoptotic neurons can also potentiate the anti-apoptotic activity of microglia [10,30], suggesting that their neurotoxic activity can be modulated by the judicious choice of modulating strategies. In general, classical pro-inflammatory cytokines (TNF-α and IL-1β) seem to be neurotoxic, whereas anti-inflammatory cytokines (IL-10) are neuroprotective [31]. Having seen that ΔRR inhibits virus-induced apoptosis in infected microglia, we wanted to know whether it also induces the production of neuroprotective cytokines. E0C and EOC20 cells were infected with ΔRR or ΔPK (moi = 5) or mock-infected with PBS and culture supernatants (conditioned media, CM) were collected at 48 hrs p.i. and UV-treated, as described in Materials and Methods, in order to inactivate any potentially remaining virus that may have escaped detection.

Primary hippocampal neurons that had been treated (or not) with NMDA (50 μM; 3 hrs) were extensively washed and the medium was replaced with a mixture of Neurobasal medium with B27 supplement and CM (1:1 ratio). Twenty-four hours later, the hippocampal neurons were assayed for apoptosis by TUNEL. The % TUNEL+ (apoptotic) cells was significantly increased in NMDA-treated than untreated hippocampal cultures (p < 0.001) and this percentage was not reduced by culture with CM from mock-infected (61 ± 2.7%) or ΔPK-infected EOC2 or EOC20 cells (45.8 ± 3.3 and 53.3 ± 4.2% respectively). CM from the ΔRR-infected EOC2 or EOC20 cells caused a significant (p < 0.001) decrease in the % TUNEL+ cells, but the decrease was significantly (p < 0.01) better for EOC20 than EOC2 cells (15.3 ± 2.7 and 25 ± 2.3 % TUNEL+ cells, respectively) (Fig. 4B). The data indicate that microglia activation by conditions other than virus infection, potentiates the ability of ICP10PK to stimulate neuroprotective modulation.

### ΔRR inhibits TNF-a production by microglia

**Figure 5:**
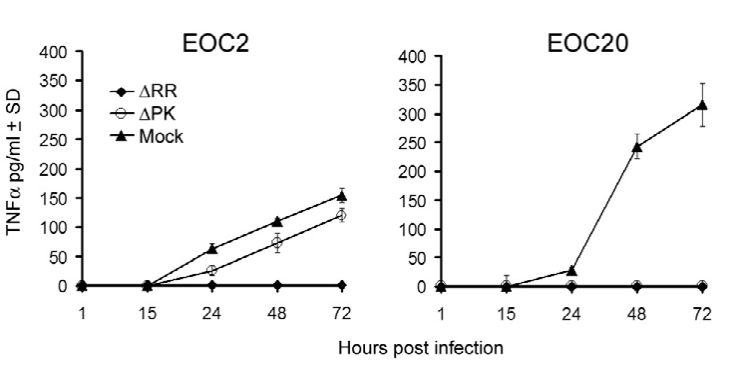
**TNF-α production is inhibited in ΔRR-infected microglia**. EOC2 and EOC20 cells were mock-infected with PBS, or infected with ΔRR, ΔPK (moi = 5) or mock-infected with PBS and culture supernatants collected 1–72 hrs p.i. were assayed for TNF-α by ELISA, as described in Materials and Methods. Results are the mean of three independent experiments ± SD. (***p < 0.001 relative to ΔRR-infected).

Having seen that CM from ΔRR- (but not ΔPK)-infected microglia protect hippocampal neurons from NMDA-induced apoptosis, we wanted to know whether neuroprotection is associated with decreased production of pro-inflammatory cytokines. We focused on TNF-α, which is a known contributor to excitotoxicity-induced neuronal cell death [5,10]. CM were collected from EOC2 and EOC20 cells infected with ΔRR or ΔPK (moi = 5) or mock-infected with PBS, at various times pi and assayed for TNF-α by ELISA. ΔPK triggered a time-dependent production of TNF-α in both EOC2 and EOC20 cells, with maximal levels seen at 72 hrs p.i. The levels of TNF-α were significantly higher for EOC20 than EOC2 cells, reaching approximately 3-fold higher concentrations at 72 hrs p.i. (315.7 ± 37.1 and 154.5 ± 12.4 pg/ml, respectively). By contrast, TNF-α was not produced in ΔRR-infected EOC20 cells, and low level production was seen in EOC2 cells (120.7 ± 12.3 pg/ml at 72 hrs p.i.) (Fig. 5). Collectively, the data indicate that ΔRR-delivered ICP10PK inhibits TNF-α production in virus-infected microglia. Inhibition appears to depend on the state of cell activation, being somewhat more potent in EOC20 than EOC2 cells.

### ΔRR inhibits RANTES production in infected EOC2 or EOC20 cells

**Figure 6:**
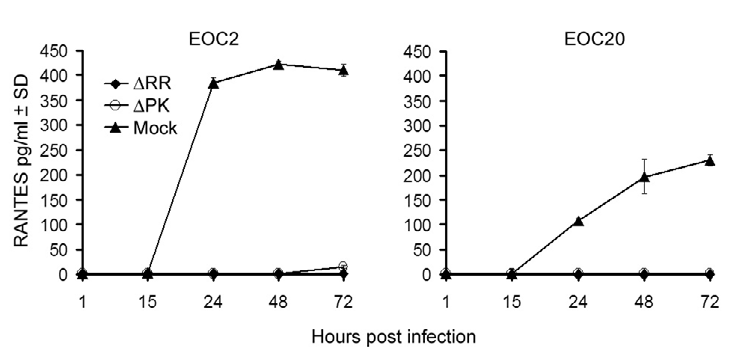
**RANTES production is inhibited in ΔRR-infected microglia**. EOC2 and EOC20 cells were mock-infected with PBS, or infected with ΔRR, ΔPK (moi = 5) or mock-infected with PBS and culture supernatants collected 1–72 hrs p.i. were assayed for RANTES by ELISA, as described in Materials and Methods. Results are the mean of three independent experiments ± SD. (***p < 0.001 relative to ΔRR-infected).

RANTES/CCL5 is a member of the C-C (β) chemokine family, which is believed to contribute to the recruitment of T cells and monocytes from the periphery into the CNS. RANTES is produced by microglia in response to pro-inflammatory stimuli [32]. Having seen that TNF-α production is inhibited in ΔRR-, but not ΔPK-infected EOC20 cells, we wanted to know whether this is also true for RANTES. Duplicate samples of the CM from the mock- or virus-infected EOC2 and EOC20 cells were assayed for RANTES by ELISA. RANTES was produced in both EOC2 and EOC20 cells infected with ΔPK. Its levels were significantly (2-fold) higher in EOC2 than EOC20 cells (Fig. 6), suggesting that its regulation is distinct from that of TNF-α. Significantly, however, RANTES was not seen in CM from ΔRR-infected EOC2 or EOC20 cells (Fig. 6), indicating that ICP10PK inhibits its production, independent of the cell activation state.

### IL-10 is produced in ΔRR-infected EOC2 and EOC20 cells

**Figure 7:**
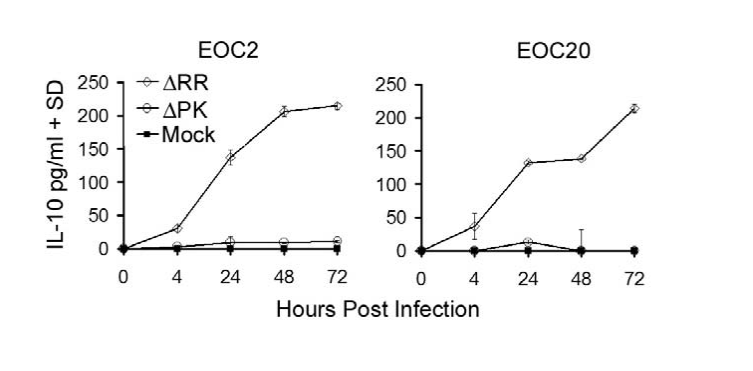
**ICP10PK induces IL-10 expression in ΔRR-infected microglia**. EOC2 and EOC20 cells were mock-infected with PBS, or infected with ΔRR, ΔPK (moi = 5) or mock-infected with PBS and culture supernatants collected 1–72 hrs p.i. were assayed for IL-10 by ELISA, as described in Materials and Methods. Results are the mean of three independent experiments ± SD. (***p < 0.001 relative to ΔRR-infected).

To examine whether ΔRR infection induces the production of neuroprotective factors and verify the effect of the cell activation state on their production, EOC2 and EOC20 cells were infected with ΔRR or ΔPK (moi = 5) or mock-infected with PBS and the CM were assayed for IL-10 production by ELISA. We focused on IL-10, because: (i) it is a pleiotropic cytokine with neuroprotective activity [31], (ii) IL-10 inhibits the transcription and translation of TNF-α and RANTES in macrophages [33], and (iii) ICP10PK upregulates IL-10 production in T cells [34]. ΔRR induced IL-10 production in both EOC2 and EOC20 cells. The kinetics of IL-10 production appeared to be somewhat different for the two cell lines, but the maximal levels at 72 hrs p.i. were similar (Fig. 7). In EOC2 cells, IL-10 was first seen at 4 hrs p.i and production increased with time, reaching maximal levels at 48–72 hrs p.i. In EOC20 cells, IL-10 was also first seen at 4 hrs p.i., but production seemed to reflect a two-phase kinetics, reaching a plateau at 24–48 hrs p.i. and increasing again, with maximal levels apparently not yet reached at 72 hrs pi. IL-10 was not seen in CM from ΔPK infected EOC2 or EOC20 cells (Fig. 7), indicating that its production is induced by ICP10PK. This is consistent with previous reports that IL-10 is not produced in microglia infected with HSV-1 [35], which does not conserve a functional ICP10PK [17,36].

### IL-10 contributes to the neuroprotective activity of the CM from ΔRR-infected EOC2 and E0C20 cells

**Figure 8:**
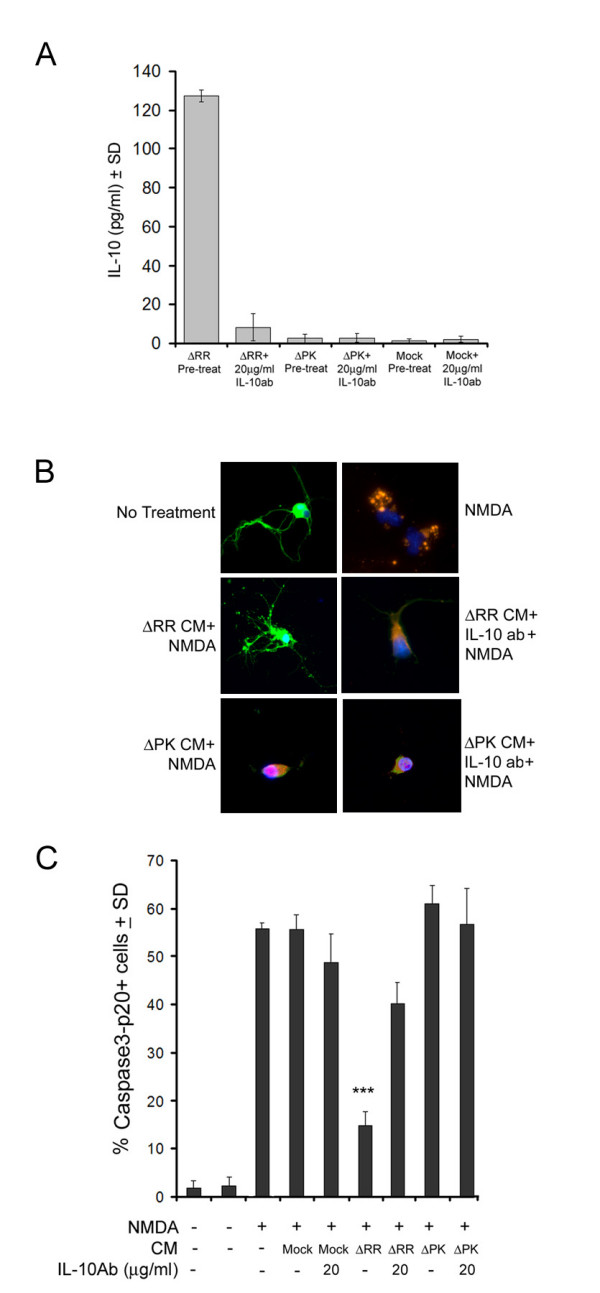
**IL-10 contributes to neuroprotection by ΔRR-infected microglia**. (A). EOC20 cells were mock infected with PBS or infected with ΔRR or ΔPK (moi = 5) and CM were collected at 48 hrs p.i. The CM were UV-treated, as described in Materials and Methods, incubated (1 hr; 37°C) with IL-10 neutralizing antibody (20 μg/ml) and assayed for IL-10 by ELISA. (B). Primary hippocampal cultures treated (3 hrs) with NMDA (50 μM) or PBS were extensively washed with MEM and re-incubated with a mixture (1:1) of Neurobasal medium containing B27 supplement and CM from infected microglia that had been treated or not with 20 μg/ml of IL-10 neutralizing antibody. They were fixed 24 h later and co-stained with AlexaFluor-546 conjugated antibody to active caspase-3 (caspase-3p20) and FITC-conjugated antibody to β_III_Tubulin (neuronal marker). Each experiment was done in triplicate and the % staining cells was determined by counting 5 randomly selected fields (at least 250 cells each, in a 3 mm^2 ^area). Results are expressed as % caspase-3p20+ cells/total number of cells determined by DAPI staining (C). ***p < 0.001, **p < 0.01 as compared to NMDA + Mock CM + IL-10 antibody.

To examine the effect of IL-10 on the neuroprotective capacity of the CM from ΔRR-infected EOC2 and EOC20 cells, we asked whether neuroprotection was lost upon IL-10 neutralization. CM obtained at 48 hrs p.i. were incubated (1 hr; 37°C) with IL-10 neutralizing antibody (20 μg/ml) and examined for: (i) IL-10 levels and (ii) neuroprotective potential in NMDA-treated hippocampal neurons, as determined by double immunofluorescent staining with antibodies to activated caspase-3 (caspase-3p20) and β III tubulin. As shown in Fig. 8 for E0C20 cells, the levels of IL-10 were significantly higher in the CM from ΔRR- than ΔPK- or mock-infected cells (127 ± 3.2, 2.8 ± 2.1 and 2.1 ± 1.5 pg/ml, respectively). IL-10 was virtually lost by neutralization (8.2 ± 6.9 pg/ml) (Fig. 8A) but its levels were not reduced by treatment with TNF-α neutralizing antibody, used as control (data not shown). The CM from ΔRR, but not ΔPK, infected cells significantly decreased NMDA-induced caspase-3 activation in hippocampal cultures. Thus, the % caspase-3p20+ hippocampal neurons (β III tubulin+) were (p < 0.001) increased by NMDA, but this increase was not seen in hippocampal cultures treated with NMDA in the presence of the CM from ΔRR-infected microglia (55.6% ± 3.0 and 14.7% ± 3.0 for mock and ΔRR, respectively). Protection was not seen in hippocampal cultures treated with NMDA together with the ΔPK CM (Fig. 8B,C). Neuroprotection, calculated as described in Materials and Methods, was 76.9 ± 5.3% for the ΔRR CM and it was reduced to 31.5 ± 7.9% by IL-10 neutralization. Neuroprotection by the mock- or ΔPK-infected CM was 3.5 ± 5.4 and -5.8 ± 6.8%, respectively. Similar results were obtained in E0C2 cells. Thus, while IL-10 contributes to neuroprotection, ICP10PK induces production of additional, as yet unidentified, neuroprotective factors and is consequently a more potent therapeutic regimen than IL-10 alone.

### ΔRR vaccination prevents KA-induced seizures and neuronal loss

**Figure 9:**
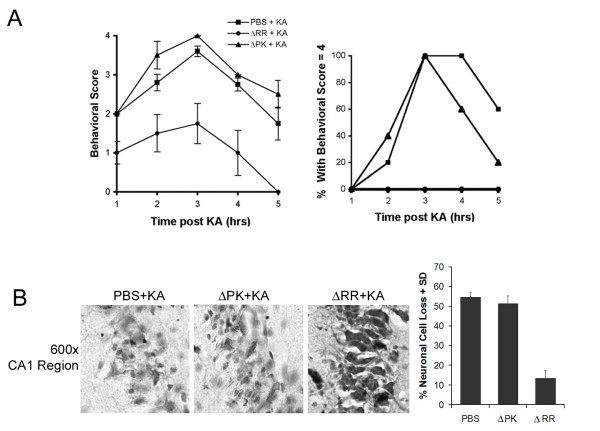
**ΔRR vaccination protects from KA-induced seizures and neuronal loss**. (A) Sprague Dawley rats were given 3 intranasal doses of ΔRR or ΔPK (5 × 10^6 ^pfu) or PBS, and given of KA (15 mg/kg) 24 hrs later by i.p. injection. They were examined for behavioral changes for 5 hours and rated on a scale of: 0, normal; 1, catatonic staring and immobilization; 2, 'wet-dog shakes', abnormal ambulation, stretching of limbs; 3, rearing and falling behavior; 4, tonic-clonic seizure activity; 5, death. Average behavioral score ± SEM is presented for each hour of observation. The % animals in each treatment group experiencing a behavioral score = 4 at any time during the observation period is shown. (B) Coronal sections of brains collected 2 days later were stained with thionin. The numbers of neurons were counted in 3 randomly selected fields of 29 μm^2 ^(at least 250 cells) from 5 serial sections for all animals and the data are expressed as % neuronal loss ± SEM relative to untreated brains.

Systemic KA injection causes epileptiform seizures, which propagate from the CA3 to the CA1 field and other limbic structures. These are followed by a pattern of neuronal cell loss, which is similar to that seen in patients with temporal lobe epilepsy [37] and is associated with microglia-related inflammatory responses [38]. We used this animal model to examine whether vaccination with ΔRR can prevent KA-induced seizures and neuronal loss. Sprague Dawley rats were given ΔRR, ΔPK or PBS intranasally and challenged with KA 24 hrs later, as described in Materials and Methods. Mock or ΔPK treated rats evidenced sustained tonic-clonic seizure activity and an increase in the associated behavioral symptoms caused by KA administration. 75% exhibited tonic-clonic seizure activity (behavioral scale = 4) at 3 hrs after KA. By contrast, ΔRR-treated animals did not progress beyond a score of 1–1.5 on the clinical scale. In the ΔRR-treated rats, symptoms completely resolved at 2 – 3 hrs after KA administration, as compared to 12 hrs in the ΔPK treated animals. While the groups averaged a score of 2 on the clinical scale, clinical response was variable, with individual animals showing severe seizures. Tonic-clonic activity was seen in 20% of the ΔPK treated rats and 40% of the PBS treated rats. Sustained tonic-clonic seizure activity and an increase in the associated behavioral symptoms were seen with time post KA administration, with 100% of the rats exhibiting tonic-clonic seizure activity (behavioral scale = 4) at 3 hrs after KA. Symptoms began to abate after 5 hours and all animals were symptom-free by 12 hrs after treatment. By contrast, ΔRR treated animals never progressed beyond a score of 1 on the clinical scale, and the symptoms completely resolved between 2 and 3 hours after KA administration. Not one of the ΔRR-treated animals displayed tonic-clonic seizure activity (Fig. 9A).

Thionin staining (recognizes the Nissl substance in live neurons) was done on the brains from the PBS- or ΔPK-treated animals that had experienced seizures with clinical scores of at least 3 and their ΔRR-treated matched pairs (clinical scores = 1 or less). Staining cells were counted in the CA1 hippocampal field, which is the recognized lesion site [26], as described in Materials and Methods. Significant neuronal loss (p < 0.001) was seen in the mock- (54 ± 1.3%) and ΔPK- (51 ± 1.9%) treated animals at 2 days after treatment with KA, but neuronal loss was not seen in the ΔRR vaccinated animals (12 ± 3.1%). Representative fields are shown in Fig. 9B.

### ΔRR-mediated neuroprotection is associated with microglial IL-10 expression

**Figure 10:**
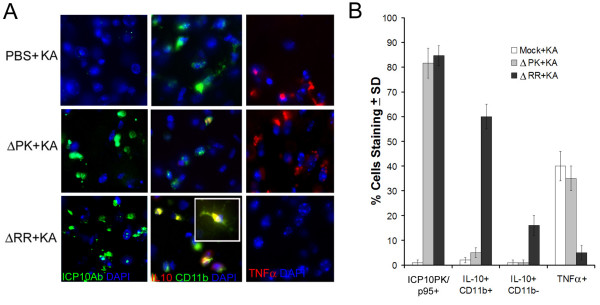
**ΔRR vaccination is associated with IL-10 production by microglia and inhibition of TNF-α**. Sprague Dawley rats were mock treated with PBS or treated with ΔRR or ΔPK [50 μl (2.5 × 10 ^6^pfu)] by intranasal delivery as described in Materials and Methods. They were given KA by i.p. injection (24 hrs later) and the brains were collected 2 days later. Serial sections were stained with FITC-labeled ICP10 antibody, Texas Red-labeled IL-10 + FITC-labeled CD11b antibodies, or Texas Red-labeled TNF-α antibody. Blue staining is DAPI. The numbers of staining cells were counted in 3 randomly selected fields of 29 μm^2 ^(at least 250 cells) from 5 serial sections for all animals and the data are expressed as % staining cells ± SEM relative to total DAPI stained cells.

To examine whether ΔRR-mediated neuroprotection is associated with IL-10 production, duplicate brain sections were stained in double immunofluorescene with antibodies to IL-10 and CD11b. Replicate sections were stained with ICP10 or TNFα antibody. ICP10PK and p95 were respectively expressed in the CA1 fields from ΔRR and ΔPK treated animals. IL-10 staining was only seen in the CA1 hippocampal fields from rats given KA and ΔRR and it primarily co-localized with CD11b (Fig. 10). Of the total CD11b+ cells in the CA1 fields from ΔRR-treated animals, 60 ± 5% also stained with IL-10 antibody. IL-10 staining was also seen in 16 ± 4% CD11b- cells, indicating that IL-10 is also produced by other cells, potentially neurons [39]. This compares to 5 ± 2% and 2 ± 1% IL-10+/CD11b+ (and no IL-10+/CD11b- cells) in the CA1 fields from rats given KA and respectively treated with ΔPK or PBS. Consistent with our results for cultured cells, TNF-α staining was barely detectable in the brains from the ΔRR treated animals (5 ± 3% TNF-α + cells), while staining was seen in the CA1 fields from animals given ΔPK (35 ± 5%) or PBS (40 ± 6%) (Fig. 10). The data indicate that ICP10PK-mediated neuroprotection is associated with microglial IL-10 production and TNF-α inhibition, as well as the production of additional, as yet unidentified, neuroprotective factors.

## Discussion

Microglia are considered the CNS resident professional macrophages. They function as the principal immune effector cells of the CNS, responding to any pathological event. Their excessive activation and the dysregulated overproduction of inflammatory cytokines are the hallmark of many neurodegenerative diseases and ischemic brain injury, emphasizing the importance of the neuronal-microglial axis [4-6,10,11]. Most of the available literature indicates that the pro-inflammatory cytokine TNF-α released by microglia activated in response to excitotoxic injury, contributes to neuronal degeneration [5,6,10,11,38,40,41]. However, microglia also produce neuroprotective factors [31], suggesting that appropriate modulation of the microglial-neuronal axis through inhibition of pro-inflammatory cytokine production and the induction of neuroprotective factors is a desirable therapeutic approach. However, identification of the target required for such microglial cell modulation and its relationship to neuronal life/death decisions, is a major clinical challenge. The salient feature of the data presented in this report is that in addition to its ability to induce survival pathways in neurons [13,17,42], ICP10PK modulates microglial responses in favor of neuroprotection, by inducing neuroprotective factors and inhibiting the production of inflammatory (neurotoxic) cytokines. The following comments seem pertinent with respect to these findings.

The construction and properties of the growth-compromised ICP10PK vector ΔRR and its ICP10PK-deleted control ΔPK, were previously described. ΔRR retains the ICP10PK gene that has anti-apoptotic activity in neurons through activation of redundant survival pathways, including MEK/ERK, PI3-K/Akt and AC/PKA [13,17,42]. ΔPK is a particularly stringent control for ΔRR because: (i) both viruses were constructed from the same HSV-2 strain and are growth-compromised in the CNS, (ii) the two viruses have no genetic differences other than the mutated ICP10 protein, as evidenced by the study of revertant viruses, (iii) the PK-deleted ICP10 protein p95 is driven by the same authentic ICP10 promoter as the mutant protein in ΔRR (p175) and both are expressed in the absence of virus replication [20], and (iv) although p175 and p95 are expressed equally well, only p175 retains kinase activity (Fig. 1). To control for the possible contribution of independent microglial cell activation (notably by excitotoxic injury) to the ΔRR neuroprotective potential, we used two cell lines (EOC2 and EOC20) that differ in their activation state. EOC20 cells constitutively express high levels of MHCII [19] and display a rounded morphology and high levels of CD11b expression before infection. This is not the case for EOC2 cells. Both cell lines were non-permissive for virus growth, but p175 and p95 (respectively encoded by ΔRR and ΔPK) were expressed, consistent with the IE regulation of the ICP10 promoter [22,23]. ICP10PK had anti-apoptotic activity, also in virus-infected microglia, and similar results were obtained in EOC2 and EOC20 cells, indicating that these properties were not affected by independent microglial cell activation.

Significantly, ICP10PK modulats the neuronal-microglial crosss-talk in favor of neuroprotection, as evidenced by the finding that CM from ΔRR-infected EOC2 and EOC20 cells protected hippocampal neurons (β_III_Tubulin+) from NMDA-induced apoptosis (determined by TUNEL and caspase-3 activation). We conclude that neuroprotection was through ICP10PK, because apoptosis was not inhibited by the CM from the ΔPK-infected EOC2 and EOC20 cells. We focused on the contribution of IL-10, because it is a pleiotropic cytokine with a strong suppressive effect on the production of pro-inflammatory cytokines by macrophages and dendritic cells [33,43], it is induced by ICP10PK in T cells from popliteal lymph nodes of virus-infected animals [34] and it has neuroprotective activity in glutamate-induced cell death or hypoxic ischemia [31]. IL-10 contributed to the ΔRR-mediated neuroprotection, as evidenced by the findings that: (i) CM from ΔRR, but not ΔPK-infected EOC2 and EOC20 cells contained relatively high levels of IL-10, and (ii) the % β_III _Tubulin+ cells (neurons) that expressed the caspase-3 cleavage product (caspase-3p20) was significantly increased by IL-10 neutralization. The effect of the IL-10 antibody was specific as evidenced by the failure to neutralize IL-10 with TNF-α, antibody (data not shown), and the failure of the IL-10 antibody to reduce the % caspase-3p20+ cells in hippocampal cultures grown with CM from mock- or ΔPK-infected microglia. However, ΔRR also induced additional neuroprotective factors, as evidenced by the finding that IL-10 neutralization did not abrogate neuroprotection and it inhibited the production of the pro-inflammatroy cytokine TNF-α and the chemokine RANTES, presumably contributing to neuroprotection through inhibition of inflammation and the recruitment of inflammatory cells to the CNS. The multiplicity of microglial effects induced by ICP10PK causes it to be a highly superior therapeutic when compared to the single use of neuroprotective cytokines, such as IL-10.

Systemic KA injection causes epileptiform seizures which propagate from the CA3 to the CA1 field and other limbic structures, and are followed by a pattern of neuronal cell loss which is similar to that seen in patients with temporal lobe epilepsy [37]. We used this model to examine the ability of ΔRR to prevent neurodegeneration, because epilepsy is a chronic disease in which periodic therapeutic dosing could prevent recurrent seizure episodes. We chose the non-invasive intranasal delivery route, because HSV gains access to the temporal lobes by the olfactory route, presumably by axonal transport [44-46], and we have previously shown that ICP10PK gains rapid (2 days) access to the hippocampus after ΔRR intranasal delivery, apparently through the lateral olfactory bulb tract [27]. However, our data did not exclude extracellular diffusion along the open intercellular clefts in the olfactory epithelium with subsequent diffusion to the olfactory bulb and CSF circulation, bypassing the blood-brain barrier [47]. Virus titers could also be minimally amplified through one round of replication in the nasal epithelial cells or other non-neuronal support cells, also causing infection of the microglia. We found that ΔRR prevented KA-induced seizures and neuronal loss in the hippocampal CA1 fields, which were associated with IL-10 production by microglia from these fields, as well as TNF-α inhibition. Unresolved questions are the mechanisms whereby: (i) ICP10PK induces IL-10 production, (ii) IL-10 protects neurons from apoptosis induced by excitotoxic injury, and (iii) ICP10PK prevents virus-induced apoptosis in microglia. Ongoing studies are designed to examine the mechanism whereby ICP10PK modulates the microglial responses. IL-10 upregulation could be related to the ability of ICP10PK to activate transcription factors, notably AP-1 [13,14,17,18,20,22,27,48] or NF-kB, and different factors could be involved in the apparent two-phase kinetics of IL-10 production in excessively activated microglial cells, such as EOC20. Similarly, ICP10PK could inhibit TNF-α and RANTES production at the transcriptional level, or inhibition could be mediated by the generation of IL-10, which is known to suppress their production in macrophages [33,49]. HSV-1, which does not retain a functional ICP10PK [17,36], does not induce IL-10 production in microglia, while triggering a vigorous cascade of pro-inflammatory responses that failed to protect susceptible mice from HSV-1-induced brain lesions [35]. HSV-1 induced TNF-α production was inhibited by exogenously supplied IL-10 [50].

Previous studies had shown that IL-10 can counteract the effect of endotoxin on cerebral metabolism in the perinatal brain [51]. However, instead of delivering IL-10 to the brain extracellular space, we can directly activate its effect as well as that of additional neuroprotective factors by using ΔRR. ΔRR has the additional therapeutic advantage that it inhibits the production of pro-inflammatory cytokines/chemokines and also functions directly in neurons, where it activates neuronal survival pathways that override apoptotic cascades [13-15,17,18,21,42]. Collectively, these functions identify ΔRR as a most promising genetic vaccine/therapy platform for neurodegenerative diseases.

## Competing interests

The author(s) declare that they have no competing interests.

## Authors' contributions

JML carried out the experiments, participated in the design of the study and the drafting of this manuscript. LA designed the vectors, conceived the experiments and drafted the manuscript. Both authors read and approved the final manuscript.
